# Pseudoxanthoma Elasticum: Cardiac Findings in Patients and Abcc6-Deficient Mouse Model

**DOI:** 10.1371/journal.pone.0068700

**Published:** 2013-07-23

**Authors:** Fabrice Prunier, Gwenola Terrien, Yannick Le Corre, Ailea L. Y. Apana, Loïc Bière, Gilles Kauffenstein, Alain Furber, Arthur A. B. Bergen, Theo G. M. F. Gorgels, Olivier Le Saux, Georges Leftheriotis, Ludovic Martin

**Affiliations:** 1 LUNAM Université, Angers, France; 2 Université Angers, Laboratoire Protection et Remodelage du Myocarde, CHU Angers, Service de Cardiologie, Angers, France; 3 CHU Angers, Centre de consultation PXE, Angers, France; 4 Université Angers, Biologie Neurovasculaire et Mitochondriale Intégrée, UMR INSERM-1083 CNRS-6214, Angers, France; 5 University of Hawai'i, John A. Burns School of Medicine, Honolulu, Hawaii, United States of America; 6 Department of Molecular and Clinical Ophthalmogenetics, The Netherlands Institute for Neuroscience (NIN), Amsterdam, The Netherlands; Scuola Superiore Sant'Anna, Italy

## Abstract

**Background:**

Pseudoxanthoma elasticum (PXE), caused by mutations in the *ABCC6* gene, is a rare multiorgan disease characterized by the mineralization and fragmentation of elastic fibers in connective tissue. Cardiac complications reportedly associated with PXE are mainly based on case reports.

**Methods:**

A cohort of 67 PXE patients was prospectively assessed. Patients underwent physical examination, electrocardiogram, transthoracic echocardiography, cardiac magnetic resonance imaging (CMR), treadmill testing, and perfusion myocardial scintigraphy (SPECT). Additionally, the hearts of a PXE mouse models (*Abcc6^−/−^*) and wild-type controls (WT) were analyzed.

**Results:**

Three patients had a history of proven coronary artery disease. In total, 40 patients underwent exercise treadmill tests, and 28 SPECT. The treadmill tests were all negative. SPECT showed mild perfusion abnormalities in two patients. Mean left ventricular (LV) dimension and function values were within the normal range. LV hypertrophy was found in 7 (10.4%) patients, though the hypertrophy etiology was unknown for 3 of those patients. Echocardiography revealed frequent but insignificant mitral and tricuspid valvulopathies. Mitral valve prolapse was present in 3 patients (4.5%). Two patients exhibited significant aortic stenosis (3.0%). While none of the functional and histological parameters diverged significantly between the *Abcc6^−/−^* and WT mice groups at age of 6 and 12 months, the 24-month-old *Abcc6^−/−^* mice developed cardiac hypertrophy without contractile dysfunction.

**Conclusions:**

Despite sporadic cases, PXE does not appear to be associated with frequent cardiac complications. However, the development of cardiac hypertrophy in the 24-month-old *Abcc6^−/−^* mice suggests that old PXE patients might be prone to developing late cardiopathy.

## Introduction

Pseudoxanthoma elasticum (PXE, OMIM #264800) is an autosomal recessive disorder characterized by the mineralization and fragmentation of elastic fibers, in the skin, retina, and vascular walls [1]–[3]. The prevalence of PXE is estimated to be between 1 in 25,000 and 1 in 50,000. Causative mutations have been identified in the *ABCC6* gene encoding an ATP-binding cassette transporter [4], [5]. The molecule(s) transported by ABCC6 presumably for systemic circulation is still unknown. As ABCC6 is primarily expressed in the liver, and to a lesser extent in kidneys, PXE is considered a metabolic disease of genetic origin [6]. The generation of an *Abcc6* knockout mouse model (*Abcc6^−/−^*) has confirmed *ABCC6* critical role in the pathogenesis of PXE [7], [8]. This mouse model recapitulates relatively well the human disease with calcifications in the ocular Bruch's membrane arterial blood vessels, kidneys but with only minor skin mineralization.

In most cases, the diagnosis of PXE is based on the combination of cutaneous and ophthalmologic findings. The main dermatological features include yellowish papules and loss of skin elasticity on the main flexural areas. Eye lesions consist of angioid streaks, which often lead to retinal hemorrhages and the loss of central vision. PXE has been associated with an increased risk of several cardiovascular complications, including hypertension, intermittent limb claudication, diastolic myocardial dysfunction, restrictive cardiomyopathy, mitral valve prolapse or stenosis, coronary artery disease, stroke, and sudden death [1], [9]–[12]. While peripheral arterial disease is commonly associated with PXE [13], several cardiac complications reportedly associated with PXE are mainly based on case reports. Therefore, the actual prevalence of cardiac complications in PXE patients is still unknown [14], [15].

Here we report the results of comprehensive cardiac investigations of a cohort of PXE patients that we compared to the cardiac findings obtained with *Abcc6^−/−^* mice.

## Methods

### Study 1: prospective study of a cohort of PXE patients

#### Patients

In total, 67 adult PXE patients were prospectively included in our referral center (PXE Consultation Center, University Hospital of Angers, France). All patients provided informed written consent, and the study was approved by our local Ethics Committee at the University Hospital of Angers (France). The study was registered at ClinicalTrials.gov (Identifier: NCT01446380).

PXE diagnosis was based on the combination of established criteria: clinically suggestive skin changes, angioid streaks, and histological demonstration of fragmented and calcified elastic fibers on skin biopsy [16].

Body height and weight measurements, medical history, physical examinations, and blood pressure measurements were routinely performed. Blood samples were collected after overnight fasting for glycemia measurement and lipid profiling. Cardiovascular risk factors were recorded for all patients: smoking habits, hypertension (*i.e.,* systolic/diastolic blood pressure >140/90 mmHg or use of antihypertensive medication), diabetes (fasting glucose ≥1.25 g/L or use of glucose-lowering medication), and hyperlipemia (low-density lipoprotein cholesterol <1.3 g/L, high-density lipoprotein cholesterol<0.40 g/L, triglyceridemia >1.5 g/L, or use of lipid-lowering medication).

All patients underwent 12-lead electrocardiography and transthoracic echocardiography. During the course of this study, several additional tests were performed to screen for potential heart abnormalities associated with PXE, such as cardiac magnetic resonance (CMR) imaging performed on 42 patients (63%), treadmill tests on 40(60%), and perfusion myocardial scintigraphy on 28(42%).

#### Echocardiography

Complete standard data from 2-dimensional echocardiography and Doppler studies was collected with a commercially-available system (VIVID7, GE Medical Systems). LV end-diastolic and end-systolic diameters and LV mass were calculated by M-mode on a parasternal long-axis view according to American Society of Echocardiography (ASE) guidelines [17]. LV hypertrophy was defined as LV mass >115 g/m2 in males and >95 g/m2 in females. LV function was estimated using the biplane Simpson's method from apical the four-chamber and two-chamber views. The severity of valve disease was classified as mild, moderate, or severe, according to ASE guidelines [18], [19].

#### Cardiac magnetic resonance imaging

CMR was performed using a 1.5T scanner (Avanto; Siemens, Erlangen, Germany) with an 8-element phased-array cardiac receiver coil. Localization was carried out using breath-hold real-time and steady-state free precession images of true anatomical axes of the heart. LV function was determined with cine imaging using a segmented steady-state free precession pulse sequence in multiple short-axis views, covering the entire LV. Late gadolinium-enhancement (LGE) was performed 7–10 minutes after administering a gadolinium-based contrast agent (cumulative dose of 0.2 mmol/kg of body weight) with a 2-dimensional segmented inversion recovery gradient-echo pulse sequence. The inversion time was individually adapted to null normal myocardium.

Commercially-available software (QmassMR 6.2.1, The Netherlands) was used for the analysis. Endocardial and epicardial borders were manually outlined on all end-diastolic and end-systolic short-axis cine slices. LV end-systolic and end-diastolic volumes, EF, and LV myocardial mass were then calculated in a standard manner. Myocardial fibrosis was defined by the presence of late gadolinium enhancement, which was evaluated under visual analysis, during which the window setting could be freely adjusted to the personal preference of the observers.

#### Exercise stress testing

PXE patients underwent a computer-assisted treadmill-exercise test according to the Bruce protocol. Cuff blood pressure measurements and standard 12-lead surface electrocardiograms were obtained in the standing position at baseline and at 3-minute intervals until the end of the recovery phase. Electrocardiograms were digitally stored and analyzed by an experienced observer. Exercise stress tests were symptom-limited or -terminated according to electrocardiographic criteria (signaled by a down-sloping or horizontal ST segment depression of at least 2 mm at 0.08 s from the J point in at least two adjacent leads). An ischemic response was defined as a horizontal or down-sloping ST-segment depression of >1 mm.

#### Single-photon emission computed tomography (SPECT)

Patients received an intravenous dose of technetium 99mTc-MIBI (3.7 MBq/kg) immediately after the exercise test. For the study under resting conditions, which was performed 4 hours after exercise stress test imaging, thrice the dose (11 MB/kg) was administered 30 minutes prior to image acquisition. Images were acquired using a 2-detector Ventri™ scintillation camera (GE Medical Systems). Images were reconstructed, and short-axis, horizontal long-axis, and vertical long-axis sections were obtained and displayed at a processing and review station, featuring a comprehensive set of cardiac applications. Technetium uptake was analyzed using a 17-segment myocardial model.

### Study 2: Heart study of Abcc6-deficient mouse model

#### Animals

All animal procedures were performed in accordance with the Guide for the Care and Use of Laboratory Animals, published by the US National Institutes of Health (NIH Publication No. 85–23, revised 1996), and approved by the Animal Care and Use Committees of the University of Angers and the University of Hawaii. The generation of *Abcc6^−/−^* mice was previously described [7]. Male *Abcc6^−/−^* mice backcrossed more than 10 times into a C57BL/6J background and their wild-type (WT) littermates of the same background were used in the study.

#### Echocardiography

Mice aged 12 and 24 months were lightly anesthetized using ketamin (60 mg/kg i.p.). Echocardiography was performed using a commercially-available ultrasound system (VIVID7, GE Medical Systems) equipped with a 13-MHz linear-array probe (IL13). All images were digitally stored on hard disk for offline analysis. Anterior wall (AW) and posterior wall (PW) thickness, LV cavity diameters, and LV shortening fraction (LVSF) were obtained from M-mode tracings recorded from the parasternal short-axis view at the level of the papillary muscles with two-dimensional image guidance [20], [21].

#### Histology

Hearts from 6-, 12- and 24-month-old *Abcc6^−/−^* and WT mice were harvested and weighed to establish heart weight over body weight (HW/BW, mg/g) ratios. Hearts were excised, fixed with formalin, and embedded in paraffin. Several sections of each heart (4–5 mm thick) were prepared, stained with picrosirius red for collagen deposition, and then visualized using light microscopy. For cardiomyocyte cross-sectional area measurements, the sections were stained with FITC-conjugated wheat germ agglutinin (WGA) from Invitrogen (Carlsbad, CA, USA) to visualize membranes of cardiomyocytes and counterstained with DAPI. The periphery of cardiomyocyte was measured using the NIH ImageJ software (http://rsb.info.nih.gov/ij/). The dimension of 100 cardiomyocytes was determined for both *Abcc6^−/−^* and WT mice groups.

### Statistical analyses

Continuous variables were presented as mean ± standard deviation (SD) in PXE patients and standard error of the mean (SEM) in mice. Categorical variables were provided as counts or absolute frequencies. Statistical analyses were performed using SPPS 15 (SPSS, Inc. Chicago, IL, USA). In Study 2, between-group differences were assessed using Student *t*-tests. *p* values <0.05 were considered as statistically significant.

The authors take responsibility for all aspects of the reliability and freedom from bias of the data presented and their discussed interpretation.

## Results

### Study 1: prospective study of a cohort of PXE patients

Table 1 summarizes the characteristics of studied cohort of PXE patients. In total, 36% (n = 24) of the patients complained of intermittent claudication. One patient with type-I diabetes underwent toe amputation. Three patients, aged 53, 61, and 64, experienced strokes. Among them, one patient had hypertension and a history of paroxystic atrial fibrillation, another one suffered from type I diabetes, and the remaining patient had hypertension. One 39-year-old individual experienced transient amaurosis related to a rare congenital carotid malformation [22].

**Table 1:** Characteristics of the study population.

Characteristics (n = 67)	
Age, years	48.3±14.9
Female, n (%)	45 (67)
Weight, kg	68.9±18.9
Height, m	1.65±0.08
Body-mass index, kg/m^2^	25.0±5.2
Diabetes, n (%)	6 (9)
Systolic blood pressure, mmHg	126±17
Diastolic blood pressure, mmHg	72±10
Heart rate, bpm	68±12
Dyslipidemia, n (%)	20 (30)
Current smoker, n (%)	12 (18)
Family history of CAD, n (%)	5 (7)
Hypertension, n (%)	19 (28)
Total cholesterol, g/L	1.81±0.33
High-density lipoprotein cholesterol, g/L	0.58±0.18
Low-density lipoprotein cholesterol, g/L	1.02±0.32
Triglycerides, g/L	1.00±0.45
Serum glucose, g/L	0.95±0.22

Data are presented as n (%), mean±standard deviation.

#### ECG findings

All patients exhibited sinus rhythm at the time of the examination. Q-waves were found in only one patient, who had a history of RCA angioplasty. A first degree atrio-ventricular block was present in one patient, a complete left-branch block was found in another, a complete right-branch block was identified in three patients, negative T waves was observed in one patient with LV hypertrophy related to severe aortic stenosis, and early repolarization syndrome was seen in two patients.

#### Coronary artery disease (CAD) investigations

As shown in Table 2, three patients had proven coronary artery disease (CAD). One 53 year-old male with cardiovascular risk factors (obesity and a family history of CAD) underwent coronary angiography due to stable angina. A single lesion was found in the right coronary artery (RCA) and treated by angioplasty and stent. The lesion was similar to those usually observed in the context of atherosclerosis. A 46-year-old smoker underwent coronary angiography because of unstable angina. A distal occlusion of the left descending coronary artery (LAD) with collateral circulation was found. This patient also had severe precapillary pulmonary hypertension of unknown origin. The last patient had undergone two venous coronary grafts at the age of 10. Prior to surgery, this young girl complained of dyspnea and atypical chest pain. A treadmill stress test had rapidly provided positive results, and coronary angiography revealed 90% mid-LAD stenosis and occlusion of proximal RCA, the lateral branches of the circumflex coronary artery, and LAD. Elastic fiber fragmentations were found on LAD biopsies. The patient is now asymptomatic after 29 years post-surgery. Computed tomography angiography performed in 2006 revealed normal functional venous coronary grafts, and dypiridamole SPECT did not find any ischemia. Three other PXE patients complained of chest pain, and all three exhibited CV risk factors, whereas treadmill tests were negative in all of them. SPECT performed in two of these patients did not show myocardial ischemia, while spastic angina was suspected in the third one.

**Table 2:** Description of PXE patients with proved or suspected coronary artery disease.

Patient	Age	Gender	CV Risk Factors	History of coronary artery disease	Results of investigations
1	60	M	BMI = 36, Familial history of CAD	Angioplasty and stent in the RCA because of exercise angina at 53 year-old	Asymptomatic, Inferior Q wave, Akinetic inferior wall on echography, Negative treadmill-test/year, Scar in 2 inferior segments on MRI
2	49	M	BMI = 28, Tobacco use	NST-ACS at 46 year-old, Distal occlusion of LAD with collateral circulation, No angioplasty	No Angina, Dyspnea because of severe precapillary pulmonary hypertension
3	39	F	None	CABG at 10 year-old because of angina due to severe LAD stenosis and RCA occlusion.	Asymptomatic, Negative scintigraphy Functional grafts on computed tomography angiography
4	73	F	Dyslipidemia, Familial history of CAD	Chest pain during exercise in 2000, Negative treadmill-test in 2000	Asymptomatic, Negative treadmill-test, Normal ECG and CMR
5	43	F	Hypertension	Chest pain at night only: spastic angina?	Negative treadmill-test and negative scintigraphy
6	40	F	Tobacco use	Atypical chest pain	Normal CMR, Normal ECG, Negative treadmill-test and negative scintigraphy

CV indicates cardiovascular; M, male; F, female; BMI, body mass index (kg/m2); NST-ACS, non ST-acute coronary syndrome; CAD, coronary artery disease, CMR, cardiac magnetic resonance imaging.

Exercise treadmill tests were performed on 40 PXE patients, combined with SPECT for 27 of them. A further patient underwent dipyridamole SPECT because of severe intermittent lower limb claudication. The peak heart rate reached during exercise testing exceeded 85% of the theoretical maximal heart rate in most patients (88%, n = 35). In two patients, peak heart rate was 65% and 72% of the theoretical maximal heart rate on account of beta-blocker intake. For one of these two patients, dipyridamole perfusion was added to the exercise test at the time of scintigraphy. Lower limb claudication has also restricted exercise tests for three of our patients. Exercise treadmill tests were all negative. In one patient, SPECT combined with the exercise treadmill test revealed perfusion abnormality in one of the 17 myocardial segments. No further investigation was performed on this patient, who was otherwise asymptomatic with normal echocardiography. Finally, dipyridamole SPECT showed abnormalities in two anterior wall segments in one diabetic patient, though his coronary angiogram was normal.

#### Myocardial and valvular findings

CMR-derived and echographic parameters of myocardial function are presented in Table 3. Mean LV volume, mass, and systolic and diastolic parameter values were all within normal range. Two patients (3%) displayed EF ≤55%, but none <50%. E/Ea ratio ≥15, indicating increased filling pressures, was observed in two patients (3%), one of which had hypertension and the other one moderate aortic valve stenosis. All patients but one (#1 in Table 2) presented normal segmental contraction. LV hypertrophy was found in seven (10.4%) patients, three of whom had a history of hypertension, and one a history of severe aortic stenosis. No etiology of hypertrophy was found in the last three cases. None of the patients presented area of myocardial fibrosis upon LGE-CMR analysis.

**Table 3:** Systolic and diastolic LV function.

Parameters	
Echocardiography	n = 67
LV end-diastolic diameter, mm	46.4±5.8
LV end-systolic diameter, mm	29.6±5.2
Diastolic anterior wall thickness, mm	8.0±1.9
Diastolic posterior wall thickness, mm	9.2±2.2
Systolic anterior wall thickness, mm	11.7±2.5
Systolic posterior wall thickness, mm	13.5±2.7
Indexed LV mass, g/m^2^	77.2±20.6
LV ejection fraction, %	64.8±6.4
E wave, m/s	0.81±0.19
A wave, m/s	0.60±0.20
E/A	1.47±0.55
E/Ea	7.5±2.89

CMR	n = 42
LV end-systolic volume, mL	47.0±14.9
LV end-diastolic volume, mL	122.9±26.1
LV ejection fraction, %	62.1±6.2
Cardiac output, L/min	5.8±1.5
Indexed LV mass, g/m^2^	71.9±22.5

LV indicates left ventricular. Data is presented as mean ± standard deviation.

Echocardiography revealed only hemodynamically-insignificant mitral and tricuspid valvulopathies (Table 4). Increased leaf thickness was found in 17 patients (25%), whereas mitral valve prolapse was detected in only three of them (4.5%). Tricuspid valve insufficiency was common (61%), but mild in all patients. Two patients exhibited significant aortic stenosis. In one 61-year-old male with a history of acute pulmonary edema, severe aortic stenosis (estimated aortic surface  = 0.7 cm2, mean gradient  = 63 mmHg) was observed. Aortic valve surgery was performed a few weeks after this finding. The second case involved a 73-year-old asymptomatic female with moderate aortic valve stenosis (estimated aortic surface  = 1.5 cm2, mean gradient  = 10 mmHg) and normal systolic function.

**Table 4:** Valve anomalies.

	n	%
Aortic valve stenosis		
Mild	0	0
Moderate	1	1.5
Severe	1	1.5
Aortic valve insufficiency		
Mild	5	7.5
Moderate	0	0
Severe	0	0
Mitral valve prolapse	3	4.5
Mitral valve insufficiency		
Mild	14	20.9
Moderate	0	0
Severe	0	0
Tricuspid valve insufficiency		
Mild	41	61.2
Moderate	0	0
Severe	0	0

### Study 2: Heart study of Abcc6-deficient mouse model

#### Echocardiography

Twenty 12 month-old mice and ten 24 month-old mice underwent echocardiography. A single 24 month-old WT mouse was excluded from echocardiographic analysis because of poor image quality.

**Figure 1:**
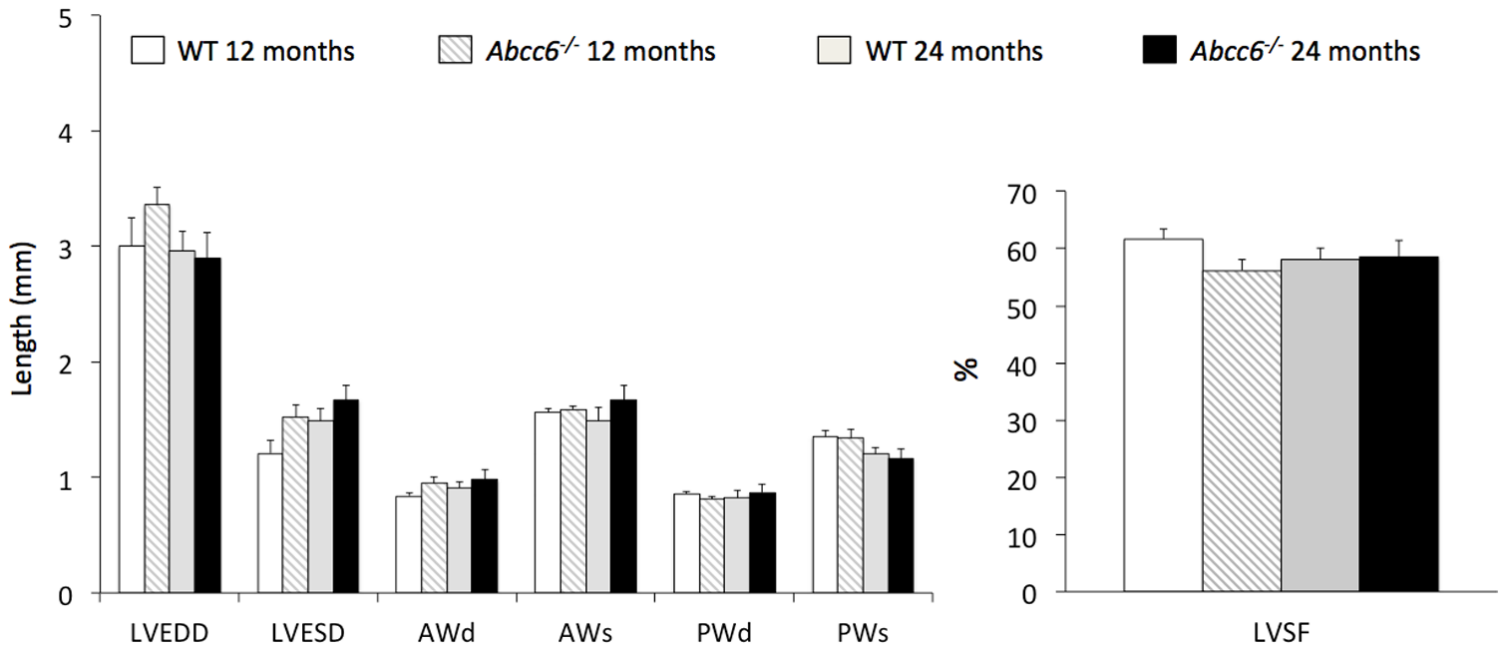
Echocardiographic data from *Abcc6^−/−^* and wild type mice. LVEDD, left ventricular end-diastolic diameter; LVESD, left ventricular end-systolic diameter; AWd, diastolic anterior wall thickness; AWs, systolic anterior wall thickness; PWd, diastolic posterior wall thickness; PWs, systolic posterior wall thickness; LVSF, left ventricular shortening fraction. WT 12 months, 12-month-old wild type mice (n = 11); *Abcc6^−/−^* 12 months, 12-month-old *Abcc6^−/−^* mice (n = 9); WT 24 months, 24-month-old WT mice (n = 5); *Abcc6^−/−^* 24 months, 24-month-old *Abcc6^−/−^* mice (n = 5). Data is expressed as mean ± SEM.

Physiological heart rates obtained at the time of echocardiography did not significantly differ between the *Abcc6^−/−^* and WT mice groups: 482±31 beats/min in *Abcc6^−/−^ vs.* 531±77 beats/min in WT, and 565±33 beats/min in *Abcc6^−/−^ vs*. 525±23 beats/min in WT, 12-month and 24-month old mice, respectively. None of the echocardiography parameters in mice 12-month- and 24-month-aged were significantly different between the *Abcc6^−/−^* and WT mice groups (Figure 1).

#### Heart weight and Histology

**Figure 2:**
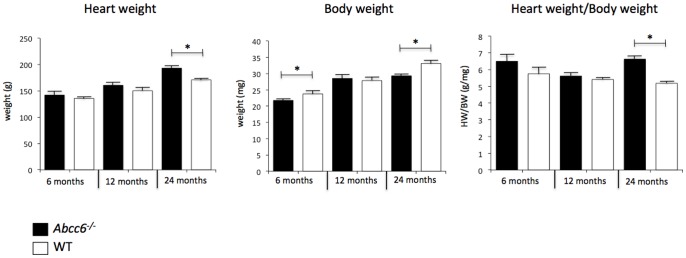
Heart and body weights. **Data is expressed as mean** ± **SEM.**

**Figure 3:**
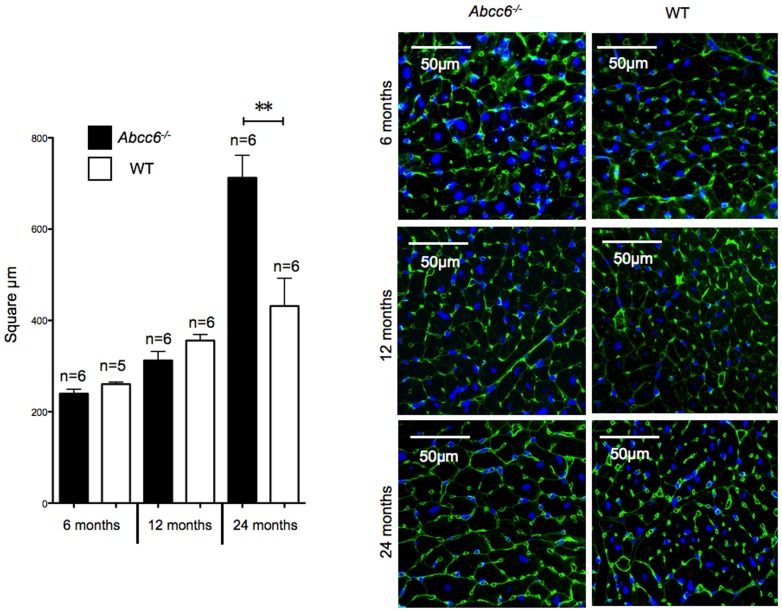
Cardiomyocyte cross-sectional area measurements. Tabulated data and representative pictures from WGA-FITC staining. All data is expressed as mean ± SEM.

**Figure 4:**
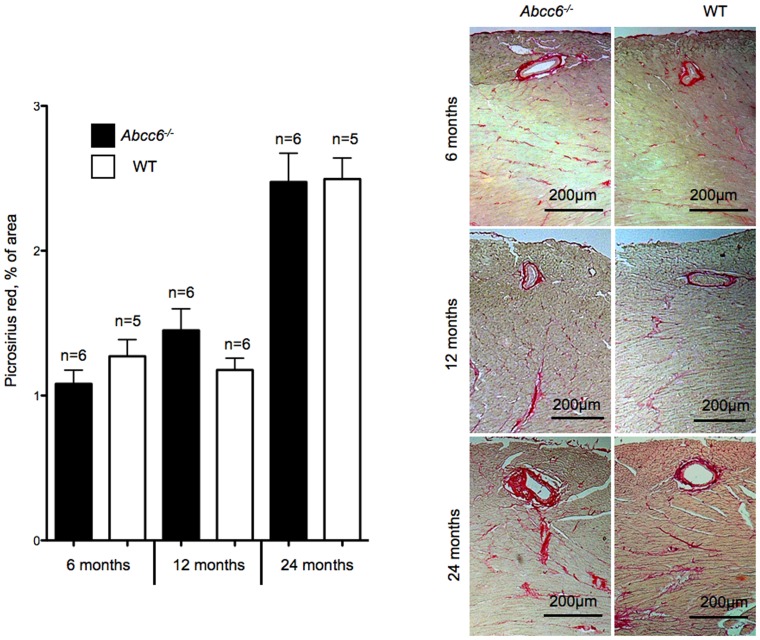
Myocardial fibrosis analysis. Tabulated data and representative pictures from picrosirius red staining. Data is expressed as mean ± SEM.

At 6 and 12 month-old, the HW and HW/BW ratios were not significantly different between *Abcc6^−/−^* and WT mice (Figure 2). WGA staining confirmed the lack of cardiomyocyte hypertrophy in *Abcc6^−/−^* mice at both ages (Figure 3) and picrosirius red staining showed the absence of any significant myocardial fibrosis in *Abcc6^−/−^* hearts (Figure 4).

Interestingly, only 24 month-old *Abcc6^−/−^* mice showed cardiac hypertrophy: the HW/BW ratios were 6.6±0.7 mg/g in *Abcc6^−/−^* mice group vs. 5.2±0.3 mg/g in WT mice group (*p*<0.01); and WGA staining revealed a clear increase in the size of cardiomyocytes (Figure 3). The cardiac hypertrophy in *Abcc6^−/−^* mice group was not associated with myocardial fibrosis (Figure 4).

In order to clarify whether the cardiac hypertrophy observed in *Abcc6^−/−^* mice group was related to higher blood pressure, systolic blood pressure was measured by the tail-cuff plethysmography method (BP2000 Series II apparatus, Bioseb, France) in a subset of trained conscious mice. Systolic blood pressure was measured daily 6 consecutive days. The results showed no significantly different between *Abcc6^−/−^* and WT mice: 115±10 mmHg in *Abcc6^−/−^* mice group vs. 118±11 mmHg in WT mice group.

## Discussion

The exact susceptibility of PXE patients to cardiac complications has never been firmly established [15]. Because some signs and symptoms of PXE overlap with common cardiovascular manifestations it has been difficult to specifically link them to PXE. Several of theses cardiac manifestations have been sporadically associated with PXE primarily in the publication of case reports. For this reason, PXE is frequently described as a condition associated with a high risk for severe cardiac events. In the present paper, we report our systematic cardiac findings in the largest cohort of PXE patients studied since the discovery of the *ABCC6* gene's responsibility for the disease and compared our results with one of the animal models currently available.

### Coronary artery disease

Severe CAD has been commonly reported in PXE cases [23]–[27]. However, myocardial infarction seems much less common than one might expect considering the arteriosclerotic lesions described in the small arterial vessels of PXE patients. Indeed, in a cohort of 100 PXE patients, Neldner identified only one patient with a previous myocardial infarction [1]. While in another study, only 2 out of 42 PXE patients exhibited prior myocardial infarction (10). Angina pectoris was present in about 13% of the patients in Neldner's study [1], which is consistent with the 12% reported by Vanakker *et al* [10]. In our cohort, three patients (4%) had verified CAD, but two of them presented cardiovascular risk factors that might have accelerated their arteriosclerosis. Severe CAD in young patients has previously been reported [26]–[28] but only one of our patients had a diagnosis of severe case of CAD in childhood. Other authors have reported extremely rare cases of sudden death occurring during active exercise in young patients [29], [30] but based on our experience, these are clearly exception to the rule.

Large PXE patient cohorts are rare, and to the best of our knowledge, prospective assessment of myocardial ischemia has never been reported. In this study, treadmill tests were normal in the 40 patients we tested. Furthermore, SPECT revealed only a minor perfusion defect in one patient out of 27, and a moderate perfusion defect in another, whom had an otherwise normal coronary angiogram. The prevalence of CAD in our cohort was at 6.0%, including the patient with a minor perfusion defect on SPECT, which is within the same range as the estimated prevalence for the European population (7.3%) between the age of 40 and 70 (mean = 52) [31].

Whether these cases of CAD are directly linked to ABCC6 deficiency is unclear but deserves further thorough investigation. Clinical findings from generalized arterial calcification of infancy (GACI [MIM 208000]), another rare autosomal-recessive disorder characterized by calcification of the internal elastic lamina, myointimal proliferation of coronary arteries, and resultant myocardial ischemia and death in young children, may support this hypothesis. Indeed GACI is associated with biallelic mutations in the *ENPP1* gene in the majority of cases, but it was recently demonstrated that *ABCC6* mutations accounted for a significant subset of GACI patients [32].

Interestingly, it has been suggested that Caucasian carriers of the most frequent *ABCC6* gene mutation (p.Arg1141X) are at an increased risk for CAD [33], [34], however, a recent study with a much larger cohort could not confirmed this data [35]. Therefore, based on the data from the few sizable cohorts published including our own, PXE does not seem to aggravate patient normal susceptibility to coronary artery disease.

### Cardiac valve disease

Fibrous thickening of the endocardium and mitral valves leading to restrictive cardiomyopathy [36], mitral valve stenosis [37], and prolapse [38], [39], have also been reported in PXE cases. Again, most of this data was based on case reports. Only two studies reported the results of systematic heart assessments by means of echocardiography in PXE patients [10], [38]. Using M-mode echography, Lebwohl M. *et al.* found mitral valve prolapse in 70% of 14 PXE patients in 1982 [38], likely reflecting the overdiagnosis of prolapse prior to the revision of diagnosis criteria and the use of the more efficient two-dimensional echocardiography. More recently, Vanakker *et al.* reported only one case (2%) of mitral valve prolapse in a cohort of 42 patients [10]. This is consistent with our data showing a limited prevalence of mitral valve prolapse in our patients (4%). Therefore, with the use of modern echocardiography, the prevalence of mitral valve prolapse in PXE patients does not appear to differ from that of the general population estimated at 2.4% [40].

Of note, similar to the data reported by Vanakker *et al.*, we frequently detected mitral valve and tricuspid valve insufficiencies in our cohort (21% and 61%, respectively), all of which were graded as “mild” [10]. Based on the Framingham population, Singh *et al.* showed that mitral and triscupid regurgitations detectable by color Doppler echocardiography were highly prevalent in the general population at more than 80% [41], which further demonstrate the absence of significant susceptibility to cardiac valve abnormality in PXE patients.

### Myocardial disease

Autopsies of PXE cases revealed direct involvement of the myocardium with endocardial lesions, characterized by degenerated elastic fibers with calcification in the subendocardium [9], [30], [39]. Congestive heart failure related to restrictive cardiomyopathy has also been reported [36]. Using standard echocardiography and tissue Doppler imaging, Nguyen *et al.* analyzed the systolic and diastolic functions of 19 PXE patients [11]. Systolic function was normal, while the means of two diastolic parameters were slightly but significantly different in PXE patients as compared with 30 healthy subjects. In our cohort, mean values of cardiac volume, mass, and systolic and diastolic parameters were within normal range. Only two patients had mild impaired EF, while two others had signs of increased LV filling pressure. Again, the previous case reports [9], [30], [39] very likely corresponded to either extremely rare cases of severe PXE or other undiagnosed conditions overlapping with PXE.

### Abcc6*^−^* ^/*−*^ mice model of PXE

*Abcc6^−/−^* mice recapitulate many of human PXE aspects, including the late onset ectopic mineralization, which becomes evident around 5–6 weeks of age [3]. As for the human disease, PXE mice develop calcification in the Bruch's membrane of retina and arterial blood vessels. The skin of these mice present very limited calcification [3], [7], [8] with the notable exception of the vibrissae that mineralize extensively and that can be used to as a biomarker to follow the progression of the disease [42], [43]. Normal cardiac dimensions and function have been reported in the same *Abcc6^−/−^* strain we used in this study [44], though these mice were much younger (10 weeks old). In the present work, we found similar LV size and function in 2-year-old *Abcc6^−/−^* mice and their controls. These results are in line with the echocardiography and CMR findings in the PXE patient cohort.

While a LV hypertrophy of unknown etiology was found in 4.5% of our PXE patients, heart weight and cardiomyocyte size were significantly increased in the 24-month-old *Abcc6^−/−^* mice. *Abcc6^−/−^* mice have normal reproductive age and life expectancy [7], [8] and at 24 month of age, they have reached about 2/ 3 of their life span. As these mice replicate rather faithfully the human condition, one might suggest that PXE patients older than 60 might be susceptible to developing late cardiopathy. It is noteworthy that the only 2 patients of our cohort older than 70 years had no evidence of cardiac hypertrophy, but one needs caution when comparing human subjects to an animal model.

Finally, Mungrue *et al.* have recently noted an increased infarct size in *Abcc6^−/−^* mice after cardiac ischemia-reperfusion [44], which may also have clinical implications for human PXE patients, though myocardial events are exceedingly rare in PXE and this fact cannot be correlated with actual cases at present.

### Limitations

In this study, we did not search for cardiac calcifications. Indeed, dystrophic cardiac calcification following a cardiac injury is a prominent feature in *Abcc6*-deficient mice [45, Brampton et al, unpublished data] even though a previous histology analysis has not found cardiac calcification in 10- to 12-week-old *Abcc6^−/−^* mice [44], after ischemia-reperfusion. The timeframe of this experiment was probably too short for calcification to develop [46].

## Conclusion

The main findings of this prospective cohort of 67 PXE patients were: 1) the low prevalence of myocardial ischemia, 2) the low prevalence of severe cardiac valve anomalies, 3) the mostly normal systolic and diastolic LV function, and 4) the lack of myocardial fibrosis. Despite sporadic counter-examples, PXE does not appear to be associated with frequent cardiac complications. However, the development of cardiac hypertrophy in the 24-month-old *Abcc6^−/−^* mice does suggest that the old PXE patients could develop late cardiopathy.
